# Labeling of Chromosomes in Cell Development and the Appearance of Monozygotic Twins

**DOI:** 10.1155/2015/628092

**Published:** 2015-06-21

**Authors:** Carol Jim, Simon Berkovich

**Affiliations:** Department of Computer Science, The George Washington University, 2121 Eye Street NW, Washington, DC 20052, USA

## Abstract

Understanding the mechanism behind the structure of the internal cellular clock can lead to advances in the knowledge of origins of pairs of monozygotic twins and higher order multiples as well as other biological phenomena. To gain insight into this mechanism, we analyze possible cell labeling schemes that model an organism's development. Our findings lead us to predict that monozygotic quadruplets are not quadruplets in the traditional sense but rather two pairs of monozygotic twins where the pairs slightly differ—a situation we coin quadruplet twins. From the considered model, the probability of monozygotic twins is found to be (1/2)^*K*^, and we discover that the probability of monozygotic quadruplets, or triplets as in the case of the death of an embryo, is (1/8)^*K*^, where *K* is a species-specific integer representing the number of pairs of homologous chromosomes. The parameter *K* may determine cancerization with a probability threshold that is approximately inversely proportional to the Hayflick limit. Exposure to some cancerization factors such as small levels of ionizing radiation and chemical pollution may not produce cancer.

## 1. Introduction

Developmental trees from an abstract mathematical perspective are useful for understanding the complex process of how a single cell develops into a multicellular organism through cell divisions and differentiation. The root of the binary tree is thought to be the zygote since it is the earliest stage of a multicellular organism's development. Cells of an organism are terminal nodes in the tree. Pairs of branches along with corresponding internal nodes represent a cell division of the preceding cell. The process of differentiating between cells is expressed by labeling the developmental tree. Tracing a path from the root to a specific cell in the tree reveals the history of its divisions.

Normal human fetal cells will divide approximately 40 to 60 times before cell division halts as demonstrated by Hayflick [1]. This phenomenon is known as the Hayflick limit. His discovery promoted the idea that each cell contains an internal cellular clock, a counting mechanism responsible for cell death and the control of cell differentiation. The reason cells eventually stop dividing is because Olovnikov [2] suggested that telomeres, sequences of DNA at the ends of chromosomes, slightly shorten with each cell division until a critical length is reached. In humans, telomeres have been implicated as a key indicator in aging as well as in cancer research. By studying the probability of monozygotic twinning (MZT), we may learn more about the structure of the internal counting mechanism and further biological implications.

Monozygotic twins form from a single zygote that divides into two separate cell masses, each giving rise to an independent individual. MZT occurs at the beginning of cell differentiation in the very early stages of development, but it is unclear as to why some zygotes split whereas others do not. Observations suggest that MZT in humans is more heavily dependent on genetic mechanisms whereas environmental factors influence dizygotic twinning (DZT) [3], where two zygotes develop at the same time into two individuals. MZT is a random event and occurs at a constant frequency in all populations globally at a rate less than that of DZT.

Much of the data on MZT frequency in various species support the hypothesis that the MZT probability is (1/2)^*K*^, where *K* is a species-specific integer. The frequency of MZT in humans is consistent at about 1 in 250 to 300 births [4, 5], which is close to (1/2)^8^. MZT data in animals is available, though scant. Berkovich and Bloom [6] and Berkovich [7] provide a comprehensive review of data on MZT frequency. In mice, it is found that MZT occurs at a frequency with 95% confidence limits of 0.2% and 2.6% [8], which gives a midrange value of 1.4%. This midrange value is close to (1/2)^6^, which is approximately 1.56%. A study on twinning and mutation rate in pigs [9] shows that, of the sampled population, MZT in pigs occurs at a rate of 1 in 525 offspring, which is close to (1/2)^9^. In a study on dairy cattle [10], data from two farms report a MZT frequency of 5.5% among all twin births and 0.33% per birth. This observed MZT frequency of 0.33% by calving event is relatively close to the frequency observed for humans at about 0.4%, which means that the probability of twinning in cattle is close to (1/2)^8^. In plants, Berkovich [11] counted the number of seeds inside the nutshell of almonds and determined that pairs, that is, two seeds inside a nutshell, occurred in about 1 in 16 cases, which corresponds to (1/2)^4^. Although there may be a different mechanism in plants, the proportion of pairs as seen in almonds still support the hypothesis of the MZT probability of (1/2)^*K*^.

Little is understood about the origins of pairs of monozygotic twins and monozygotic higher order multiples such as triplets and quadruplets in both humans and animals. In this paper, we provide some insight into the mechanisms involved in twinning by investigating cell division labeling from the considered hypothetical cell labeling model. In addition to twinning, these same mechanisms may provide some implications into cancerogenesis. The significance of telomeres on the human aging process is also considered. Both the aging process and cancerogenesis have been noteworthy topics under extensive study currently. The reasons why humans age and the cause of aging have been questioned but no concrete answers exist. Likewise, the process of certain cells becoming cancerous and methods to halt this process are still being investigated. From our considered model, the mechanisms involved in the two opposite circumstances of twinning and cancerogenesis may provide a foundation for the understanding of the origins of these two disparate processes.

## 2. Model Design

### 2.1. Possible Cell Labeling Models

Cell labeling schemes of a developmental tree can be used to depict the cell development of an evolving organism. Two possible cell labeling models of increasing complexity are analyzed and considered: the binary cell labeling scheme and the chromosomes labeling scheme. We focus on the chromosomes labeling scheme to derive the possible cell labeling combinations and conduct our investigation into the mechanisms involved in twinning and cancerogenesis.

#### 2.1.1. Binary Cell Labeling Scheme

**Figure 1:**
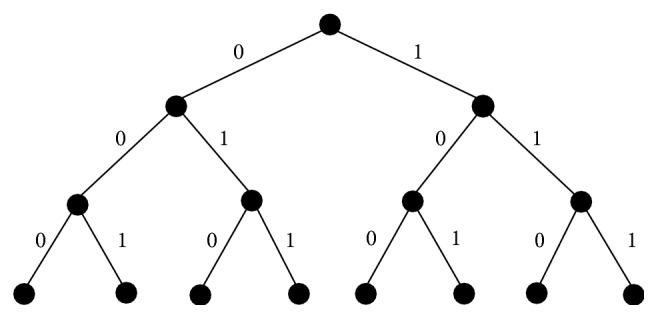
Binary cell labeling scheme.

When applied to biological systems, development models need to show how cell differentiation occurs where a single cell will eventually produce multiple cells that differ in its functionality after self-reproduction. Each cell divides into two individual cells with both of these new cells not necessarily being identical, thus creating a binary tree. The binary cell labeling scheme involves labeling the binary tree with 0's and 1's as shown in Figure 1, which illustrates the first three cell divisions. Each node in the tree represents a cell. In this abstract scheme, its materialization by telomeres is considered, hence the binary labeling of the newly created cells. This scheme does not, however, take into consideration any other further complexities of labeling. For example, it does not distinguish between the cells all labeled as “0” and between the cells all labeled as “1.” Rather, the scheme uses the sequence of 0's and 1's of the path from the root to a certain cell in the tree to make the distinction between cells. The binary sequence also describes the history of the cell's origin.

The binary cell labeling scheme illustrates the process of cell differentiation. Berkovich [12] explains that this scheme provides decentralized organization and cell system control for a developing system where control is spread across the entire population. A class of cells comprises all cells with a common descendent. A class of cells, for example, cells containing the sequence 10*XX* ⋯ *X*, may be differentiated into one type whereas a class of cells within a larger class of cells, for example, cells containing the sequence 100*X* ⋯ *X*, may be differentiated into a subtype. The symbol *X* may represent either a “0” or a “1,” and the types and subtypes that the classes of cells differentiate into may become various tissues and cell types such as muscle cells, skin cells, and nerve cells.

#### 2.1.2. Chromosomes Labeling Scheme

The chromosomes labeling scheme expands upon the binary cell labeling scheme to take into account DNA changes during replication as well as the asymmetry of cells during an organism's development. Rather than simple binary choices in cell labeling as in the binary cell labeling scheme, the chromosomes labeling scheme exhibits a more sophisticated cell labeling procedure due to the semiconservative replication process. Before a cell divides, it must replicate its chromosomes so that the predetermined instructions carried by DNA can be passed onto the daughter cells when cell division occurs. During replication, a chromosome's two DNA strands unwind and separate and proceed through semiconservative replication where a complementary strand is produced for each original strand. A short piece of RNA is needed to begin the process of creating the complementary strands. Telomeres shorten after each cell division because of the space that is taken up by this piece of RNA, thereby causing the new complementary strands to be slightly shorter in length as compared to the original strands.

**Figure 2:**
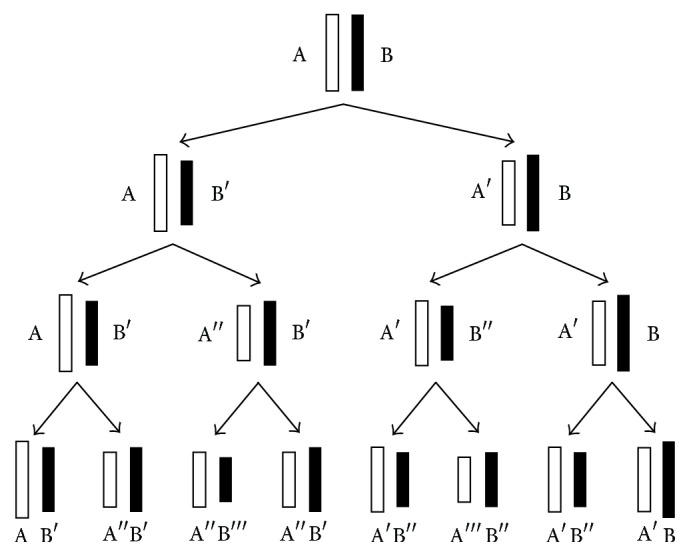
Chromosomes labeling scheme of the cellular clock model [11].

A hypothetical model of the internal cellular clock that takes into account these chromosomal changes during semiconservative replication is suggested by Berkovich [11]. This chromosome labeling scheme for the first three cell divisions along with a cell labeling mechanism is illustrated in Figure 2. The root of the tree consists of a chromosome in the original cell that contains two original strands of DNA, that is, the two complementary halves of the double helix structure, labeled as AB. In the first division and during the semiconservative replication process, the two original strands of DNA labeled as AB will separate and a complementary strand is produced for A (which we label as B′) and another complementary strand is produced for B (which we label as A′). Thus, two daughter cells will form after the first division containing DNA labeled as AB′ and A′B, where ′ represents a newly formed complementary strand. These newly formed strands are slightly shorter than the original strands because of telomere shortening after the cell division. Each of these daughter cells has a combination of one original DNA strand and one newly synthesized strand, which we will define to be a hybrid strand. Hence, both cells contain hybrid strands after the first division. During the second division, the process continues with the hybrid strands separating and a new complementary strand is synthesized for each strand. These new cells contain DNA labeled as AB′, A′′B′, A′B′′, and A′B. Thus, half of the cells contain hybrid strands whereas the other half contain completely new strands after the second division. This process of cell division and labeling continues where every subsequent division will result in fewer hybrid strands and more completely new strands [13]. The pattern of labeling in the model is such that if a single strand accumulates *i* changes, then its complement can only have (*i* − 1) or (*i* + 1) changes as noted by Berkovich in [11].

### 2.2. Cell Labeling Combinations

**Figure 3:**
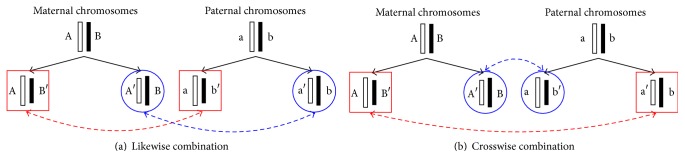
Two possible cell labeling combinations: (a) likewise combination and (b) crosswise combination.

Various possible cell labeling combinations can be deduced by using the chromosomes labeling scheme. Since humans are diploid organisms, each cell contains pairs of homologous chromosomes where each homologous pair contains one chromosome from the mother (maternal chromosome) and one from the father (paternal chromosome). Homologous chromosomes are similar since each contains the same genes in the same order; however, they are not identical because the alleles for each trait may be different. In the course of division, the daughter cells receive random, independent combinations of chromosome copies from the parent cells.  Figure 3 shows the two possible combinations that can occur: a likewise combination and a crosswise combination. A likewise combination pairs the left labeling of the maternal and paternal subtrees together and pairs the right labeling of both subtrees together. A crosswise combination pairs the left labeling of the maternal subtree with the right labeling of the paternal subtree together and also pairs the right labeling of the maternal subtree with the left labeling of the paternal subtree together.

**Figure 4:**
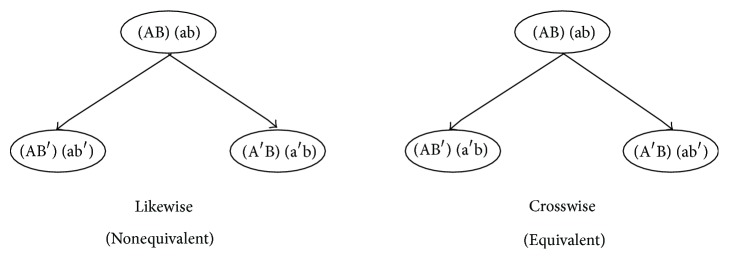
Cell labeling of possible homologous chromosomes combinations at the initial zygote division [6].

Pairs of homologous chromosomes control the cellular clock mechanism. As an example, we can label a pair of homologous chromosomes in the initial state of the zygote at the root of the tree as [(AB), (ab)]. Consequently, the cell labeling associated with the two possible combinations after the first division can be represented by Figure 4 from Berkovich and Bloom [6]. The states of the internal cellular clock are dependent upon the combinations of the chromosome changes as division occurs. Assuming homologous chromosomes influence the states of the internal clock in a similar way, equivalent labeling can be achieved. In the labeling, we represent maternal chromosomes with capital lettering and paternal chromosomes with lowercase lettering. Therefore, the components of the homologous chromosomes are equivalent where A ≡ a and B ≡ b. In this manner, the crosswise combination in Figure 4 will give equivalent labeling for the two offspring.

If we assume that *K* pairs of homologous chromosomes control the internal cellular clock, then there is a possibility of 2^*K*^ different ways of the organism beginning its development. This is due to the zygote's chromosomes being passed on during its division independently. As previously noted, the two offspring in the crosswise combination will exhibit equivalent labeling, that is, identical states of the internal cellular clock, out of the two possible states of the organism at the initial zygote division. Thus, the probability of such an occurrence for MZT, *P*(MZT), is

(1) $$\begin{array}{c} P\left(\;MZT\;\right)={\left(\;\frac{1}{2}\;\right)}^{K}, \end{array}$$

where* K* indicates number of pairs of homologous chromosomes. Globally, the frequency of human monozygotic twins is constant at a rate of approximately 1 in 250 to 300 births [4, 5]. The probability of (1/2)^8^ falls into that range, suggesting that some set of 8 pairs of homologous chromosomes are essential in the control of a human zygote's development [11].

## 3. Results and Discussion

### 3.1. Appearance of Quadruplet Twins as Identical Pairs

By investigating the cellular labeling using the chromosome labeling scheme, we can see the appearance of MZT and possible higher order multiples. The simplest situation of MZT occurrence is in the case of the crosswise combination at the initial zygote division. The equivalent labeling of the offspring shows the nonadvancement of the internal cellular clock. When this occurs, a cell systems' control is distributed across both cells rather than centralized within the zygote, and the process of organism development has not yet begun. Each of these offspring will begin with identical states in their internal cellular clocks and eventually develop into two distinct organisms to create identical twins. Because of the rarity of twinning events and higher order multiples, decentralized cell systems' control is less likely to occur as compared to centralized control in the zygote. Besides MZT occurrences, we focus our attention on the possibility of higher order monozygotic multiples after the initial zygote division.

**Figure 5:**
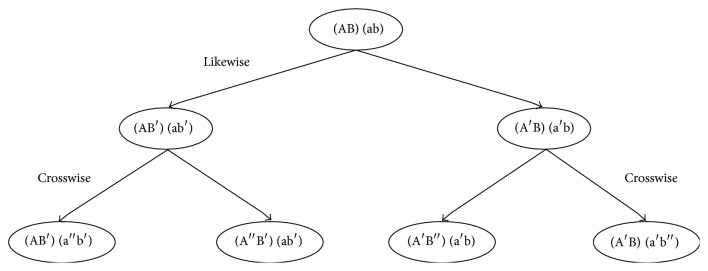
Quadruplet twins appearance in the second cell division.

We observe the first appearance of quadruplets when investigating the second cell division. The quadruplets in this case are two pairs of monozygotic twins with the cells in each pair having identical states of the cellular clock as seen in Figure 5. We coin the term “quadruplet twins” hereafter to describe this type of situation. Unlike multizygotic, or fraternal, quadruplets where the four offspring are the result of four separate egg and sperm combinations, the observed quadruplet twins are monozygotic quadruplets. The offspring are the result of one zygote splitting into two embryos, each of which further splits again. If one of the quadruplet offspring happens to die, we may see the formation of triplets.

In the second cell division, there is a possibility of eight states, that is, labeling combinations of homologous chromosomes, of the organism. Because of this, we discover that the probability of the occurrence of monozygotic quadruplets (or triplets), *P*(MZQ), is

(2) $$\begin{array}{c} P\left(\;MZQ\;\right)={\left(\;\frac{1}{8}\;\right)}^{K}, \end{array}$$

where *K* is defined above. Assuming *K* = 8 as previously found for humans, the probability of human quadruplet births, or triplets if one offspring dies, is (1/8)^8^. This is equivalent to a frequency of 1 in about 16 million, which is close to the odds that are observed in [14–17]. It is estimated that only about 60 sets of identical quadruplets exist worldwide [15, 17, 18]. Because of the extreme rarity of identical triplets, quadruplets, and other higher order multiples, it is difficult to accurately calculate their frequency and find this data in scientific literature. Instead, it can usually be found in the common press when experts weigh in on the rarity of such an occurrence.

Based on our findings, monozygotic quadruplets in humans may actually be two pairs of monozygotic twins where one pair may be slightly different than the other. This prediction of the theory can be seen by our cell labeling mechanism of homologous chromosomes. The cells of each pair of quadruplet twins have identical states of the internal cellular clock whereas the two pairs slightly differ in their genetic labeling. Our proposed theory that monozygotic quadruplets are different pairs of monozygotic twins can easily be tested by comparing the DNA of the quadruplet twins at various points on the genome and determining if any differences exist. It would be expected that the two pairs of twins in a set of quadruplet twins will have some slight differences in their genome, but individuals within each pair will have more or less the same genomic structure.

It has been observed in a few instances where monozygotic twins are identical but have some noticeable distinctions due to differing genomes rather than environmental factors. Previous studies [19, 20] have suggested that epigenetic factors, that is, changes in gene expression due to chemical modification, are the cause of the differences between monozygotic twins. However, genetic variation has been shown to play an important role in making identical twins different in recent studies [21, 22]. Somatic mutations such as copy number variants or errors where a twin's DNA has a different number of copies of a particular gene at certain points in the genome can occur early in the development of the fetus. Such a situation may arise in the case of quadruplet twins causing the pairs of twins to be slightly different. If two of the twins die, then the indicated situation occurs.

Taking into consideration another organism other than humans, the nine-banded armadillo and five other closely related species in the genus* Dasypus* regularly exhibit the phenomenon of monozygotic quadruplet births. These six species of armadillo evolved to ensure reproductive success by consistently producing genetically identical quadruplets with every litter to bypass the physical constraints of the uterus [5, 23]. The identical quadruplets are formed similar to the structure seen in the second cell division of our chromosome labeling scheme. The embryo splits and each of the resulting cells further splits again. Our prediction of the theory on monozygotic quadruplets in humans may also apply to other species such as the armadillo. Quadruplets in the aforementioned six species of armadillo may not be quadruplets in the traditional sense but rather two different pairs of monozygotic twins like the occurrence seen in Figure 5. This may be true for armadillos in South and Central America due to the fact that armadillos in Brazil have been found to have more genetic variability than the nine-banded armadillo in the United States based on the study in [23]. However, the prediction of the theory may not apply to the nine-banded armadillo in the United States where offspring in a quadruplet litter are genetic clones of one another. This is because armadillos originated from South America, and the nine-banded armadillo is the only species of armadillo found outside of South and Central America. Therefore, they are adapting to unusual conditions in their recent arrival to the United States.

Since the nine-banded armadillo is the only frequently studied species and no accurate population counts exist, it is difficult to determine the actual frequency of quadruplet births across all species of armadillos. There are currently a total of 20 living species of armadillos, and it is known that the six species of armadillos in the genus* Dasypus* always give birth to monozygotic quadruplets whereas species in other genera of armadillos usually have a litter of one offspring [23]. However, let us assume that the population of all species of armadillos is equal in ratio. Then, the frequency of monozygotic quadruplets in armadillos is 6 in 20 births. The closest *K* value to fit formula (2) for this frequency is* K* = 1 as determined by 6/20 = 3/10 ~ 1/8 = (1/8)^1^. This suggests that one pair of homologous chromosomes controls the zygote's development in armadillos.

### 3.2. Implications of Telomeres on Aging and Cancerogenesis

Telomeres help prevent the strands of DNA on the ends of chromosomes from unraveling, and they naturally shorten as cells divide. There has been much debate on whether telomere shortening causes aging or if aging causes telomere shortening, if at all. The common hypothesis is that telomere shortening contributes to human aging. However, literature reviews on this topic [24, 25] show that evidence supporting this claim is ambiguous. Not only is telomere shortening with age in cells difficult to measure, but data on telomere length across a life span in various tissues is lacking. There is also a lot of variation in average telomere length amongst same-aged individuals and amongst different species of organisms. Determining which telomere length measure, for example, longitudinal change or average telomere length, is most informative is another important deciding factor in determining if a relationship between telomeres and aging exists. All of the above factors contribute to the difficulty in establishing a causal relationship between telomeres and aging.

Despite all of these uncertainties, it is known that telomeres play a significant role in the cellular response to traumas such as stress and DNA damage [25]. Short telomeres are associated with many different complications including illness, cellular aging, diseases of premature aging, and other chronic diseases. Therefore, telomere shortening increases a human's susceptibility to various environmental, physical, and psychological stressors rather than causing “normal” aging in humans. It is possible that as telomeres shorten, cell sensitivity to DNA damage increases making these damage signals replication independent. Telomeres may also affect cell differentiation instead of aging since many different factors besides solely telomeres seem to contribute to the human aging process.

A recent study [26] reports that people who drink more regular soda have shorter telomeres in their white blood cells and thus accelerated cellular aging. Daily consumption of a 20-ounce soda was calculated to be equivalent to a telomere shortening of an average of 4.6 years. Researchers determined no link between diet sodas and fruit juices and cellular aging, however. They cautioned that the association between telomere length and sugar-sweetened soda consumption does not demonstrate causation since these two data points were only compared for each participant at a single time point. Furthermore, it is unknown whether lifestyle factors among the sample of adults or other variables may have influenced the telomere length of the individuals during the length of the study.

On the other hand, another recent small pilot study [27] following men who are in the early stages of prostate cancer shows that telomeres may actually lengthen over time by implementing some lifestyle changes. A healthy diet, moderate exercise, stress reduction, and social support all led to increased telomere length by about 10% in men in the test group whereas men in the control group saw a decrease in telomere length by about 3% after the end of the five-year study. The healthy lifestyle changes seem to increase telomerase activity causing the increases in telomere length which may help prevent certain chronic diseases.

The enzyme telomerase, which is expressed by germ cells and certain stem cells but not by most human somatic cells, reverses telomere shortening and extends the replication potential of cells. Cells that express telomerase still experience cellular senescence, that is, the cessation to divide, in response to certain toxins, oncogenes, or DNA damage. Previously, it is thought that cellular senescence appears as a function of chronological age; however, the majority of recent studies that measured in vitro replicative capacity as a function of donor age fail to support this correlation [28]. Cellular senescence is believed to occur from changes in molecular structures that either result from deterioration or are preprogrammed. In principle, such changes are reversible. This inspires gerontology to a specific course of action in cellular research. However, at the system level, the limitations of the lifespan of biological organisms must involve some factor of irreversibility. The system control including biological memory should incorporate an indispensable algorithm for resolution of multiple responses to ensure content-addressable access.

In the case of the Big Data situation arising in biological information processing, the pivotal operation of resolution of multiple responses is performed by a certain stream algorithm [29]. The selection in this stream algorithm is determined by a prevalent fraction of replicated data. This fraction will fall below an operational threshold since memory expands as the time passes. As a result, the control functions of biological information processing degrade irrevocably. The same algorithmic circumstance—low fraction of replication—deprives infants of future recollections of the events of their early life. The reason, however, is opposite: this fraction is low not because the denominator, the size of the memory, is large, but because the numerator, the extent of replication, is small. Thus, the irreversibility of aging at the end of life and some restrictions at the beginning of life are related; a famous French proverb says:* “les extrêmes se touchent”* (total opposites come into contact).

Although the internal cellular clock and the concept of the Hayflick limit can be seen in humans and in most mammalian cells, this may not be the universal case across all animal species depending on some factors. Most notably, laboratory mice do not exhibit progressive telomere shortening due to long telomeres and telomerase activity [30]. Thus, the idea of the Hayflick limit occurring because of telomere shortening does not apply to mice, although this is the case as seen in humans. Rather, the behavior of certain cell types as if they are evading the Hayflick limit without reaching replicative senescence is an exception to particular types of cells and occurs under specific culture conditions. For example, the study in [31] claims that most rat oligodendrocyte precursor cells from the optic nerve are able to evade replicative senescence and do not stop dividing. This, however, only occurs under specific culture conditions that prevent cells from differentiating and avoid activation of responses that arrest the cell cycle. In a separate study [32], some mouse cells have been found to behave as if they do not have a Hayflick limit when grown in 3% oxygen concentration levels as opposed to 20% oxygen. More DNA damage was seen in the mouse cells in 20% oxygen than in 3% oxygen.

Despite certain cells in mice dividing indefinitely under certain laboratory conditions, laboratory mice cells still have a Hayflick limit, albeit lower than that of humans [33]. It is explained in [34] that mice cells become senescent after 10 to 15 mitotic divisions in vitro and that mice have a short lifespan of about two years. Thus, the Hayflick limit is not determined by telomere shortening in mice but rather may be due to DNA damage in the cells. The universality of the Hayflick limit is dependent upon the type of cell studied and environmental conditions. Humans are naturally exposed to various environmental factors that may increase telomere shortening whereas laboratory mice are held in highly controlled and sterile environments which may disrupt effects of interactions among factors affecting telomere erosion [34]. Generally, it has been found that telomere length inversely correlates with lifespan in mammals, and telomerase expression inversely correlates with body size [30, 34]. This indicates that telomeres have different purposes depending on the species.

Apoptosis, that is, programmed cell death, and the occurrence of Hayflick's limit are essential for the stabilization of constancy of cell agglomerations in working organisms. Remarkably, insight towards the process of cancerogenesis can be seen by both the considered mechanism of MZT at the beginning of cell development and apoptosis at the end. The emergence of cancerogenesis is a result of a breakdown of protection by apoptosis. Namely, the probability of failure of the cell death, *Z*_*T*_, between cell divisions is given by the formula from [11]:

(3) $$\begin{array}{c} Z_{T}≃\frac{\ln\left(3-2/K\right)}{M+\alpha \sqrt{M}}\sim \frac{1}{M}. \end{array}$$

It is approximately inversely proportional to the Hayflick limit *M*. Here,* K* is the species-specific integer parameter representing the number of pairs of homologous chromosomes, and *α* is a standard deviation coefficient about 1. Equation (3) assumes that the Hayflick limit for a species is known regardless of whether the Hayflick limit is determined by telomere shortening as in the case of most mammals or by some other factor such as DNA damage as seen in the case of laboratory mice.

If some cancerization factor, such as exposure to radiation or pollution, is to exceed a certain threshold, cancer results. Otherwise, the critical level for the formation of malignancy will not be reached by the number of potentially malicious cells that escape apoptosis. The parameters of the phenomenon of MZT in certain laboratory animals, like mice, can be used to study the considered threshold effect for cancerization. The results can then be quantitatively transferred and compared with the observations on humans. Consequently, the parameter *K* derived from our cell labeling of the chromosomes labeling scheme can be considered in the two opposite circumstances of MZT and cancerogenesis.

The epigenetic clock developed by Horvath [35] may provide more insight into the causes of aging in addition to cancer research by measuring the biological age of organs and tissues using DNA methylation levels. He provides a proof of concept showing that the biological clock can be reset to zero by reprogramming adult cells back into a stem-cell-like state. Nevertheless, uncovering the mechanism behind the internal cellular clock, of which we provided insight into, is the key to future developments in developmental biology, the human aging process, and cancerogenesis.

## 4. Conclusions

By investigating the chromosomes labeling scheme and possible cell labeling combinations, we discover that monozygotic quadruplets may be two pairs of monozygotic twins where the pairs of twins are slightly different from one another. This theory can be tested by DNA analysis of the twin pairs and may apply to not only humans but animals like the armadillo as well. The investigation into twinning provides a foundation for understanding the process of cell development through which the cell development mechanism is established. The parameter *K* reveals the structure of the internal cellular clock and determines the threshold effect in cancerization. For example, cancer may occur if the intensity of radiation exposure exceeds a certain level. This theory can be tested on laboratory animals such as mice where *K* = 6. The Hayflick limit and cellular senescence act as an additional protection from cancer [36]. Without it, cancer is more likely to occur but is not inevitable. The process of apoptosis has been found to have a genetic basis where factors that control cell proliferation and differentiation can affect cell numbers, and mutations can disrupt cell death leading to cancer and other human diseases [37]. The phenomenon of biological aging may be to a considerable degree associated with irreversible factors in view of the complications in the organization of Big Data information processing.
